# Knowledge, attitudes and perceptions of pharmacy and nursing students towards male circumcision and HIV in a KwaZulu-Natal University, South Africa

**DOI:** 10.4102/phcfm.v4i1.327

**Published:** 2012-07-11

**Authors:** Panjasaram V. Naidoo, Farzana Dawood, Christine Driver, Magdalene Narainsamy, Sikhanyiso Ndlovu, Victor Ndlovu

**Affiliations:** 1 School of Pharmacy and Pharmacology, University of KwaZulu-Natal, South Africa

## Abstract

**Background:**

Male circumcision is currently being promoted in South Africa as a Human Immunodeficiency Virus (HIV) prevention method. Effective implementation requires that healthcare providers should believe in the procedure's efficacy and should possess a positive attitude. A study was undertaken amongst pharmacy and nursing students with different objectives.

**Objectives:**

To ascertain students’ knowledge, attitudes and perceptions regarding male circumcision and (HIV) prevention.

**Method:**

A descriptive cross-sectional study using anonymous questionnaires was undertaken amongst 4th year pharmacy and nursing students studying at a university in KwaZulu-Natal, after obtaining their consent. Data were captured and analysed using SPSS version 15.

**Results:**

A response rate of 83.18% and a mean knowledge score of 66.43% with relatively positive attitudes (62.7) were obtained; 85.4% of the respondents felt that promoting male circumcision is appropriate, with all Muslim students (*n* < 11) supporting the promotion of male circumcision. Even though all Muslim students supported male circumcision, only 3 students were willing to perform the procedure if adequately trained (*p* < 0.03). The majority of the female students were unwilling to perform the procedure (*p* < 0.005). A third of the respondents indicated that male circumcision would both undermine existing protective behaviours and strategies as well as increase riskier sexual behaviour. Over 54% of the respondents believed that the South African Health System would be able to cope with the massive male circumcision drive. The majority of the respondents favoured the procedure to be done at birth. Pain was cited as the most important reason for not wanting to be circumcised.

**Conclusion:**

Pharmacy and nursing students have a moderate knowledge of male circumcision and HIV prevention with relatively positive attitudes. The majority felt that promoting male circumcision is appropriate and should be encouraged.

## Abstrait

**Connaissances, attitudes et perceptions des étudiants en pharmacie et en écoles d'infirmières relatives à la circoncision masculine**

**Présentation:**

La circoncision masculine est actuellement promue en Afrique du Sud comme méthode de prévention du virus d'Immunodéficience acquise (VIH). Une bonne mise en œuvre de cette méthode nécessite que les prestataires de soins de santé croient en l'efficacité de l'opération et aient une attitude positive à cet égard. Une étude a été réalisée chez les étudiants en pharmacie et en écoles d'infirmières avec différents objectifs.

**Objectifs:**

Vérifier les connaissances, les attitudes et les perceptions des étudiants quant à la circoncision masculine et la prévention du (VIH).

**Méthode:**

Une étude transversale descriptive utilisant des questionnaires anonymes a été réalisée chez des étudiants en 4è année de pharmacie et d’école d'infirmière dans une université du KwaZulu-Natal, après avoir obtenu leur consentement. Les données ont été saisies et analysées par SPSS version 15.

**Résultats:**

Un taux de réponse de 83.18% et une note de connaissances moyenne de 66.43% avec des attitudes relativement positives (62.7) ont été obtenus; 85.4% des étudiants interrogés avaient le sentiment qu'il était approprié de faire la promotion de la circoncision masculine, tous les étudiants musulmans (*n* < 11) soutenant la promotion de la circoncision masculine. Bien que tous les étudiants musulmans aient été en faveur de la circoncision masculine, seuls trois étudiants étaient prêts à réaliser l'opération s'ils recevaient la formation adéquate (*p* < 0.03). La majorité des étudiants de sexe féminin ne souhaitaient pas réaliser l'opération (*p* < 0.005). Un tiers des interrogés ont indiqué que la circoncision masculine saperait les comportements et stratégies de protection existants et augmenterait les comportements sexuels à risque. Plus de 54% des interrogés considéraient que le système de santé sud-africain ne pourrait faire face à l’élan massif de circoncision masculine. La majorité des interrogés étaient en faveur d'une opération à la naissance. La douleur était citée comme la principale raison pour ne pas vouloir se faire circoncire.

**Conclusion:**

Les étudiants en pharmacie et école d'infirmière disposaient d'une connaissance moyenne de la circoncision masculine en rapport avec la prévention du VIH, et faisaient preuve d'attitudes relativement positives à cet égard. La majorité avait le sentiment qu'il était approprié de promouvoir la circoncision masculine et que celle-ci devrait être encouragée.

## Introduction

### Key focus

The number of people living with Human Immunodeficiency Virus (HIV)has risen from approximately 8 million in the year 1990 to 33 million infections today.^1^ Sub-Saharan Africa is the region in the world most affected by HIV and AIDS with 22.4 million people living with the virus in the year ending 2008.^1^ By mid-year 2009 an estimated 5.2 million people were infected with HIV in South Africa with the province of KwaZulu-Natal having the highest prevalence rate of HIV infections as depicted in the antenatal clinic attendees’ data.^1^ Due to the enormity of HIV infections present and growing in South Africa today, various preventative strategies have been devised and are currently being used to curb the transmission rate of HIV.^2^ The methods used include prevention of mother to child transmission (PMTCT), AIDS awareness campaigns, condom use and distribution, the promotion of abstinence, and the limitation to one sexual partner.^2^ Recently another method has been introduced, namely male circumcision. Male circumcision is a new strategy, only receiving World Health Organization (WHO) recognition in March 2007.^3, 4^

### Trends

Scientific studies have shown that circumcised men are up to 50% – 60% less likely to acquire HIV during heterosexual intercourse.^5, 6, 7^ Other studies have shown that HIV infection rates are highest in traditionally non-circumcising areas of sub-Saharan Africa^8^. Infection rates have been less significant amongst populations that traditionally practice male circumcision than in communities where the procedure is rare.^6^ However, the WHO clearly stated that male circumcision was not ‘a magic bullet’ and should not replace any of the established methods of HIV prevention.^5^ The WHO further advised that promoting male circumcision ‘should be recognized as an additional, important strategy for the prevention of heterosexually acquired HIV infection in men’.^4, 6^ At the WHO/UNAIDS Technical Consultation on Male Circumcision and HIV Prevention in Montreux, Switzerland, it was recommended that countries with high hyper endemic and general HIV epidemics should consider scaling up access to male circumcision services as a priority for adolescents, young men and older men who are at a higher risk of HIV infection.^4^ Male circumcision used to be carried out in the Zulu culture (the predominant ethnic group in the province of KwaZulu-Natal) but was suspended more than 200 years ago by the Zulu king, Shaka Zulu, because septic wounds from the circumcision procedure had left many men unable to participate in the war.^3^ In January 2010 the premier of KwaZulu-Natal stated that he would embark on a massive male circumcision programme before the end of the year with the help of healthcare professionals.^3^ Apart from physicians who would be performing the circumcisions on patients, there seemed to be an indication that other healthcare professionals would assist in the programme.^3^ According to the scope of practice for nurses in South Africa as set out by the South African Nursing Council (SANC), nurses are involved in the following acts: ‘the prevention of disease and the promotion of health by teaching to and counselling with individuals and groups of persons’ and ‘preparation for and assistance with operative, diagnostic and therapeutic acts for the patient’.^9^ Therefore it can be expected that nurses could be actively involved in the new male circumcision programme.

The South African government's strategic plan for HIV and AIDS, which is enforced by the South African Pharmacy Council (SAPC), compels pharmacists in South Africa to play an active role in the prevention, treatment, care and support of HIV patients^10^. It was stipulated that pharmacists should play a greater role in distributing AIDS-related information to the public, educating the public on promotion and therapy of sexually transmitted infections (STIs) and HIV, and promoting an understanding of the disease amongst members of the public.^10^ Therefore it can be expected that pharmacists would be greatly involved in the newly introduced male circumcision programme, informing members of the public about the procedure, promoting male circumcision and by providing treatment, care and support to male circumcision patients.

These two categories of health professionals in particular, whose scope of practice is directly aligned with the management of HIV patients, should ensure that they have the necessary skills in HIV prevention treatment and support to help curb the epidemic.

### Objectives

In addition, for male circumcision to be implemented successfully as part of South Africa's HIV prevention strategy, it is important that the health care providers themselves believe in the procedure's efficacy and possess a positive attitude toward the procedure. It is only then that male circumcision promotion would be achieved sustainably.

### Research significance

For male circumcision programmes to succeed and be firmly embraced and recognised as part of HIV prevention in South Africa, it is important that pharmacy and nursing students (i.e. future healthcare professionals) have an adequate knowledge on HIV and male circumcision. This would probably lead them to having a positive attitude.^11, 12^ It was therefore decided to undertake a study with pharmacy and nursing students in order to determine their knowledge, attitudes and perceptions in terms of male circumcision with regard to HIV.

## Ethical considerations

Permission was sought from the Dean of the Faculty and the heads of the School of Nursing and Pharmacy (FECHSC 010/10). Prior to completing the questionnaire, the study was explained to the students and those willing to participate signed a consent form. Participation was voluntary and withdrawal from the study was allowed at any time. Confidentiality and anonymity was ensured by coding all questionnaires. No student was forced or coerced to participate in the study.

## Method

### Design, setting and sample

A descriptive cross-sectional study was conducted at the University of KwaZulu-Natal with the use of an anonymous close ended questionnaire amongst final year nursing and pharmacy students. A list of all the registered final year Pharmacy and Nursing students which totalled 107 students was obtained from the Faculty Officer of Health Science. The variables in the questionnaire included the demographic profile, knowledge, attitudes and perceptions toward male circumcisions as well as willingness to perform the procedure. Prior to administration the questionnaire was pilot tested by staff members of the pharmacy department.

### Analysing

Data were captured and statistically analysed using statistical package for social science (SPSS) version 15. Analysis of variance (ANOVA), frequency and percentage ratios, Chi-square and *t*-tests were utilised in the statistical analysis. Bonferroni Tests had been applied as Post-Hoc tests for Oneway ANOVA analysis. In all analyses, the usual significance level was 0.05.

Possible responses to knowledge questions pertaining to male circumcision with regard to HIV were ‘*yes’, ‘no’* and *‘unsure’*. Each correct answer in the knowledge section of the questionnaire was credited with 1 point, whereas wrong and unsure answers were disregarded. The total possible score in the knowledge section was 8 points. This section consisted of questions on HIV and male circumcision's effect on HIV. The knowledge score was obtained by summing up the individual questions. For all questions that measured knowledge, a higher score represented better knowledge. Attitude and perception sections were combined to form the second section. The score for this section was evaluated in a similar manner to the knowledge score, where each positive response to a question was credited with 1 point. The total possible score in this section was 11.

## Results

A total of 89 students completed the questionnaire, giving a response rate of 83.18%. A summary of the demographic characteristics of the study subjects is presented in Table 1.

**TABLE 1:** Socio-demographic data of 66 pharmacy and 23 nursing students.

Variable	*n*	%
**Gender**		
Male	26	29.2
Female	63	70.8
**Age**		
19–21	37	41.6
22–24	38	42.7
25–28	9	10.1
29–31	3	3.4
>31	2	2.2
**Race**		
African	57	64.0
Indian	30	33.7
Other	2	2.2
**Religion**		
Christian	61	68.5
Hindu	17	19.1
Muslim	11	12.4

*n*, Given as number of participants.

Total number of respondents = 89

There were more female respondents than male respondents with the majority of the respondents between the ages of 19 to 24 (Table 1).

### Knowledge on HIV and male circumcision

An overall mean knowledge score of 5.31 was obtained which translated to 66.43% (s.d.+/-17.43). There was no substantial difference between the knowledge of pharmacy students as compared to the nursing students. Pharmacy students obtained a mean knowledge score of 5.27 (*n* = 66, 65.9%) and nursing students scored an average of 5.48 (*n* = 23, 68.48%).

Over 92% per cent of the respondents knew that male circumcision is not as effective as condom use alone at preventing HIV transmission and that one must still wear a condom during sexual intercourse, even if one were circumcised. However, less than 45% of the respondents were aware that male circumcision decreases HIV transmission (40.5%) and that it is easier for an uncircumcised male to acquire HIV (42.7%). A greater proportion of nursing students (47.8%) compared to pharmacy students (37.9%) knew that HIV can survive longer in the foreskin of the penis.)

**TABLE 2:** Students’ knowledge on HIV and male circumcision with regard to pharmacy and nursing students.

Variables	Correct				*N*	%

	Pharmacy		Nursing			
	
	*n*	%	*n*	%		
What does HIV stand for?	66	100	23	100	89	100
What does AIDS stand for?	60	90.9	20	87.0	80	89.9
1. Do you think male circumcision will decrease HIV transmission?	29	43.9	7	30.4	36	40.5
2. Do you think HIV can survive longer in the foreskin of the penis?	25	37.9	11	47.8	36	40.5
3. Do you think by removing the foreskin, the penis will become tougher and more resistant to HIV and STI?	22	33.3	6	26.1	28	31.5
4. Is it easier for an uncircumcised man to acquire HIV?	23	4.8	15	65.2	38	42.7
5. Do you think that male circumcision is as effective as condom use alone at preventing HIV transmission?	61	92.4	21	91.4	82	92.1
If one were circumcised, would one still have to wear a condom during sexual intercourse?	62	93.9	23	100	85	95.5

*n*, Given as number of participants.

Pharmacy students, *n* = 66; nursing students, *n* = 23; total number of students, *n* = 89.

AIDS, acquired immune deficiency syndrome; HIV, human immunodeficiency virus; STI, sexually transmitted disease.

### Attitudes and perceptions

The mean attitude score for the respondents was 62.7 (*n* = 89) (s.d.+/- 18.54).

As can be seen in Table 3, 85.4% of the respondents indicated that promoting male circumcision is good whilst 60% of the respondents perceived that promoting male circumcision would cause an increase in riskier sexual behaviour. Whilst almost a third (31%) of the respondents, indicated that male circumcision would both undermine existing protective behaviours and strategies as well as increase riskier sexual behaviour. Further just over 50% of the respondents felt that the South African Healthcare System would be able to cope with the introduction of a massive male circumcision campaign.

**TABLE 3:** Attitudes and perceptions of pharmacy and nursing students in terms of male circumcision and HIV.

Variables	Positive Response				Combined Positive Response	
	
	Pharmacy		Nursing			
		
	*n*	%	*n*	%	*n*	%
Women would want their male partners to be circumcised.	38	57.6	9	39.1	47	85.5 (*n* = 55)
Promoting male circumcision is good.	51	77.3	19	82.6	70	85. (*n* = 82)
Whether or not they would undergo a circumcision, assuming the procedure is safe.	9	13.6	9	39.1	18	69.2 (*n* = 26)
An HIV infected man should be circumcised.	31	47.0	14	60.9	45	59.2 (*n* = 76)
Promoting male circumcision would not cause an increase in riskier sexual behaviour.	28	42.4	6	26.1	34	39.1 (*n* = 87)
Male circumcision will not completely prevent HIV from being spread.	65	98.5	21	91.3	86	96.6 (*n* = 89)
Male circumcision aids in the prevention of HIV.	22	33.3	7	30.4	29	33.0 (*n* = 52)
Male circumcision reduces the man's risk of contracting HIV during penetrative sex.	35	53.0	12	52.2	47	56.0 (*n* = 84)
Would the South African health system cope with the massive male circumcision drive?	39	59.1	9	39.1	48	54.5 (*n* = 88)
Having unprotected penetrative sex with a circumcised man will not prevent a woman from contracting HIV.	56	84.9	21	91.3	77	88.5 (*n* = 87)
Male circumcision will undermine existing protective behaviours and strategies.	33	50.0	7	30.4	40	44.9 (*n* = 89)
Male circumcision would make a marked difference in South Africa's fight against HIV.	38	57.6	12	52.2	50	60.2 (*n* = 83)

*n*, Given as number of participants.

AIDS, acquired immune deficiency syndrome; HIV, human immunodeficiency virus; STI, sexually transmitted disease.

**TABLE 4:** Barriers cited by male respondents for not wanting to be circumcised.

Barrier to undergoing circumcision	*n*	%
Pain	5	62.5
Cost	0	0
Against religion	2	25.0
Against culture	1	12.5
No trust in South African health system	0	0
Fear of death	4	50.0

*n*, Given as number of participants; *n* = 8.

Of the 26 male respondents, 18 (69.2%) stated that they would undergo circumcision if the procedure was safe. Pain was cited as the most common barrier by those male respondents who did not want to have a circumcision.

Respondents indicated that the procedure should be performed on newborn children (Table 5).

**TABLE 5:** Preferred age for circumcision procedure.

Age	Discipline				Total	

	Pharmacy		Nursing			
		
	*n*	%	*n*	%	*N*	%
1. Newborn	37	60.7	15	75.0	52	64.2
2. Puberty	21	34.4	5	25.0	26	32.1
3. Adulthood	3	4.9	0	0	3	3.7

*p* = 0.390

*n*, Given as number of participants; *n* = 81.

All the respondents belonging to the Islamic faith supported the promotion of male circumcision. The findings were not statistically significant (Table 6).

**TABLE 6:** Perception in terms of promoting male circumcision regarding respondents’ religious denomination.

Religion	Promoting male circumcision is good		*N*	%

	*N*	%		
Christian	47	(82.5)	57	69.5
Hindu	12	(85.7)	14	17.1
Muslim	11	(100)	11	13.4

*p* = 0.321

*N*, Total per religious group; *n* = 82.

### Willingness to perform circumcisions

Just under 50% of the respondents, that is 49.4% (*n* = 89), expressed a willingness to perform male circumcision if they were adequately trained; however, when this collection of data was further broken down according to the religious affiliations of the students, a significant finding was that only 3 of the 11 Islamic students were willing to perform the procedure (*p* < 0.03). Only 60.9% of the nursing students (*n* = 23) showed a willingness to carry out circumcisions. Further significant data obtained indicated that a greater percentage of those unwilling to perform the procedure were female respondents (*n* = 45, 84.4%, *p* < 0.005).

## Discussion

A moderate knowledge score for pharmacy and nursing students in their final year of study and studying at a university that is situated in the province with the highest HIV prevalence rate does not auger well for the epidemic and its management by future health care professionals. A possible reason for this relatively moderate score pertaining to male circumcision knowledge could be that male circumcision is a relatively new concept in HIV prevention, only being formally recognised and accepted by the WHO in 2007 ^4^; hence, it was probably not included in the present HIV curriculum that is being taught at the university. Of concern, however, is that students did not show knowledge on current evidence based methods of prevention relating to HIV and AIDS as less than 41% knew that male circumcision will decrease HIV transmission. Numerous studies have shown that mass media (i.e. newspaper, magazines, television and radio) is a major source of providing information about HIV and AIDS.^13, 14, 15^ Male circumcision promotional advertising in mass media in South Africa has been scarce and much less prominent when compared to the other prevention strategies such as PMTCT, condom usage, and abstinence. Although articles supporting male circumcision have been published on news websites and in newspapers, highly visible media advertising campaigns have been lacking. This could be another reason why male circumcision knowledge is moderate amongst the students in the study.

Pharmacists act as important sources of health advice and medical information to members of the public, with many people turning first to their pharmacists for medical advice.^16, 17^ The successful implementation of new health strategies or programmes is directly influenced by provider preparedness and user acceptability.^18^ Both these points further emphasise the importance of nurses and pharmacists being adequately informed and educated on male circumcision with regard to HIV so that they will be sufficiently equipped to advise enquiring members of the public.

As acknowledged by Mantell et al.^18^ when discussing the introduction of the female condom in South Africa, the successful implementation of the female condom would be achieved, only when health care providers themselves believe that the female condom is acceptable and efficacious. They would then be able to ‘sell’ the preventive strategy as a viable alternative to their patients.^18^ It is thought by the researchers that a similar analogy can be drawn to the introduction of male circumcision in South Africa as a preventive strategy against HIV. In order for male circumcision to be implemented successfully as part of South Africa's HIV prevention strategy, it is important that the health care providers themselves believe in the procedure's efficacy and possess a positive attitude to the procedure. It is only then that male circumcision promotion would be achieved sustainably.

Although it was widely acknowledged by the students that male circumcision is not as effective as condom use and that condoms still ought to be worn, the majority of respondents did think that promoting male circumcision is good. Whether or not they would promote circumcision, however, is unknown. As reported by Castro et al.^19^ medical providers who are not involved in circumcision campaigns and who have lower knowledge pertaining to the procedure are often reluctant to discuss it with their patients. This would suggest that modifying the educational content to include more about male circumcision is essential for South African healthcare workers.

An equally moderate score for attitudes could directly relate to the knowledge. As suggested by a number of studies, individual respondent's attitudes become positive especially after being educated on the topic.^12^ It was found in our study that the respondents displayed relatively positive responses toward the questions asked. It can be deduced that if the respondents had prior training and additional knowledge on male circumcision, they would have been more confident in answering the questions and would have achieved an even higher attitude score. Moreover, some students hold negative attitudes and risk perceptions that could become barriers in their eventual professional treatment of patients. Respondents showing negative attitudes towards the procedure could be explained by not clearly understanding the overall benefits of male circumcision. The respondents’ backgrounds may significantly influence their attitude, because certain cultures deem circumcising as promoting bad behaviours.^20, 21, 22^

Many interventions in educational and practical programmes in different parts of the world have shown promising outcomes with regard to caring for HIV patients.^11^ This could be an approach with nursing and pharmacy students. In a study done in Turkey, it was shown that the educational preparation of nurses had affected the attitudes of the nurse and the effectiveness of the care provided to the patient living with HIV.^12^

The perception that male circumcision would increase riskier sexual behaviour and undermine existing preventative strategies adds on to the many concerns that people have whenever a new method of prevention, treatment or care relating to HIV is introduced. Once men are circumcised, they may be under the impression that they are protected from contracting HIV, and thus will compensate for their perceived risk reduction by engaging in higher risk behaviours. A moderate level of risk compensation could mitigate any benefit of circumcision in preventing HIV infections. Some observational studies have found that circumcised men engage in higher risk behaviours than uncircumcised men.^20, 23^

Some people felt that males would not use condoms anymore; however, it has to be borne in mind that these strategies are not for exclusive use but should be used to complement each other. Circumcision will be most effective if it is not perceived as a stand-alone clinical procedure, but as one component, and should be delivered as a part of a recommended package of HIV prevention and reproductive health services. This should include: HIV testing and counselling, information about the risks and benefits of male circumcision, condom promotion, behavioural change counselling and promotion, and the management of Sexual Transimmited Infections (STI). Therefore careful monitoring and evaluation of providing male circumcision as a service must be ensured.^4^ Massive educational campaigns should be carried out whenever new methods of interventions are being mooted in order to reinforce appropriate and continued non-risky sexual behaviours.

The age at which a man can be circumcised also has to be considered. When the respondents were asked what the recommended age for circumcision should be, the majority felt the procedure should be done as a newborn. The *Children's Act Section 12(8)(b)* expressly allows for circumcision of men under the age of 16 under specific circumstances;^24^ it states that circumcision is prohibited except for religious purposes or medical reasons.^24^

In the absence of a definition of ‘medical reasons’, and based on the evidence that male circumcision reduces the risk of HIV infection by up to 60%, and that neo-natal circumcision carries a lower risk of complications than childhood or adult circumcisions^25^, it is proposed by the researchers that the interpretation of ‘medical reasons’ be broad enough for a medical practitioner to recommend elective neo-natal medical male circumcision to the parents of infant boys and thus remains within the ambit of the *Children's Act*.

In a previous study it was found that male circumcision was strongly associated with religious variables.^26^ It was seen that a greater percentage of the population who were Muslim was strongly associated with more male circumcision prevalence as compared to a predominantly Christian population.^26^ Of the 11 Muslim students who participated in our study, all indicated that they felt promoting male circumcision is good. This is most likely due to the fact that in Islam, male circumcision is carried out when the child is born and is considered compulsory. Thus the religion may have played a role with these students’ responses. It can therefore be inferred that health care providers who have a cultural or religious background that supports circumcision would very likely promote the procedure to those who are non-circumcised.

Pain was cited as the most common barrier to respondents not wanting to be circumcised. This finding is consistent with other studies where it was found that the apprehension of pain during and after the procedure was reported to be the major barrier to male circumcision acceptability.^20^

Half the respondents indicated a willingness to perform male circumcisions. This could be attributed to their moderate knowledge in terms of the procedure. Women were less willing to carry out male circumcisions, which could be attributed to women being less comfortable than men in carrying out procedures which involve male sex organs.^27^

Even though the nurses showed a higher willingness to perform male circumcisions due to the possibility that non-invasive or minor surgical procedures are already done by nurses in hospital and clinic settings, all the nurses were not willing. In KwaZulu-Natal the premier is expecting other health professionals to assist with the procedure and with the absence of defining a health professional; it may be possible that he is referring to a nurse as they are directly involved with doctors in the operating theatre.^9^ In addition, the male circumcision procedure practiced in the KwaZulu-Natal programme makes use of the Tara Klamp, which is a non-invasive technique.^28, 29^ Therefore it is recommended that a further study be conducted with practising nurses in the province to gauge their willingness to perform the male circumcision procedure and to identify the barriers that make them unwilling.

### Limitations of the study

Time constraints and convenience was a limitation to the study in that it was difficult to synchronise a convenient time for the participating students and the researchers as the study had to be explained during lecture time which resulted in a loss in lecture time or insufficient time to explain and motivate the participation of students. A further limitation of the study was the small sample size.

## Recommendations

The schools of nursing and pharmacy should re-examine their curriculum with the intent of including content on male circumcision as a preventive strategy in HIV management. National health authorities should increase public awareness in terms of the beneficial effects that male circumcision has on HIV prevention. A further study should be done that includes gauging the attitudes and perceptions of currently practising nurses toward male circumcision and their willingness to perform the procedure.

## Conclusion

Nurses and pharmacists were found to have moderate knowledge and relatively positive attitudes regarding male circumcision and HIV and AIDS. There was also unwillingness by certain nursing and pharmacy students to carry out the procedure. Relatively positive attitudes regarding male circumcision suggest that with proper education and training, these health care professionals would be willing to perform the procedure as well as promote and educate patients on matters regarding male circumcision. With greater education, some of the perceptions shared by the respondents could be corrected.
